# Postoperative visits by dedicated anesthesiologists in patients with elevated troponin: a retrospective cohort study evaluating postoperative care utility and early detection of complications

**DOI:** 10.1186/s13741-020-00152-6

**Published:** 2020-07-16

**Authors:** Annemarie Akkermans, Lisette M. Vernooij, Wilton A. van Klei, Judith A. van Waes

**Affiliations:** 1 Department of Anesthesiology, University Medical Centre Utrecht, Utrecht University, Utrecht, the Netherlands; 2 Department of Epidemiology, Julius Centre for Health Sciences and Primary Care, University Medical Centre Utrecht, Utrecht University, Utrecht, The Netherlands

**Keywords:** Troponin, Perioperative anesthesiologist, Screening, Myocardial infarction

## Abstract

**Background:**

An elevated cardiac troponin level after noncardiac surgery is associated with both morbidity and mortality. Guidelines suggest routine troponin monitoring in high-risk patients. We implemented a dedicated anesthesia team to conduct follow-up on patients with postoperative troponin elevation. We hypothesized that these visits would facilitate early detection of complications. Therefore, the aim of this study was to evaluate the effect of postoperative visits by dedicated anesthesiologists on early detection of complications and care utility.

**Methods:**

This retrospective observational study included patients aged ≥ 60 years with an elevated troponin within the first 3 days after noncardiac surgery. Troponin elevation was detected by routine biomarker monitoring. The primary outcome was early detected myocardial infarctions by the dedicated anesthesiologist. Other outcomes were overall detected complications, additional diagnostic tests and treatment advised by the anesthesiologist, consultation of another medical specialist, and advised postoperative follow-up at the outpatient cardiac clinic within 1 week after surgery.

**Results:**

Of the 811 patients, 509 (63%) received a postoperative consultation by the anesthesiologist. Anesthesiologists were involved in the early detection of 59% of all myocardial infarctions and in 12% of all complications. Besides cardiac ischemia, patients were also often diagnosed with noncardiac complications, including respiratory failure (8.9%), pneumonia (13.2%), and acute kidney injury (17.5%) within 1 week after surgery. In 75% of patients, anesthesiologists ordered additional diagnostics, most frequently existing of electrocardiograms and additional cardiac enzyme testing. Additionally, change in treatment was advised, most often a medication change, in 16% of patients.

**Conclusions:**

Standard consultation of a dedicated anesthesiologist resulted in an early detection of 59% of all myocardial infarctions and involved a change in treatment in a considerable number of patients with postoperative troponin elevation. Whether this may improve patient outcomes remains to be elucidated.

## Introduction

An increased serum cardiac troponin level, as a marker for postoperative myocardial injury, has been shown to be an independent predictor of morbidity and mortality within the first year after non-cardiac surgery (Devereaux et al. 2017; Levy et al. 2011; van Waes et al. 2016; van Waes et al. 2013). Depending on the amount of troponin elevation and the surgical population, the incidence of mortality within 30 days varies between 4 and 17% (Devereaux et al. 2017; Devereaux et al. 2012; van Waes et al. 2013). As only 15% of patients with postoperative myocardial injury experiences typical ischemic symptoms (Devereaux et al. 2011), routine postoperative troponin surveillance is recommended by several guidelines for postoperative risk stratification to direct secondary prevention (Duceppe et al. 2017; Thygesen et al. 2018).

In our center, we implemented a routine postoperative troponin I (TnI) surveillance program in January 2011. In a previous study in which this program was evaluated, it became apparent that only 41% of all patients with postoperative myocardial injury received cardiac consultation (van Waes et al. 2016). Cardiac etiology was suspected in almost half of the consulted patients. However, troponin elevation has also been related to other disease entities including stroke, sepsis, and pulmonary embolism (Akkermans et al. 2019; Beattie et al. 2018; Noordzij et al. 2015; van Waes et al. 2016). We hypothesized that a more general approach to assess postoperative troponin elevation, rather than an approach focusing on cardiac etiology, would be beneficial. As a result, in October 2016, we implemented routine postoperative consultations by a dedicated anesthesia team in patients detected with an elevated troponin in the surveillance program. The goal of these visits was to improve postoperative follow-up and to potentially detect or prevent complications at an early stage. We hypothesized that such routine visits would facilitate early detection of complications with only a limited use of resources. Therefore, this study aimed to evaluate the effect of these visits by dedicated anesthesiologists on early detection of complications and postoperative care utility in patients with troponin elevation after noncardiac surgery.

## Methods

This study was conducted in adherence to the STROBE statement for observational research (von Elm et al. 2008). The local Research Ethics Committee assessed the study protocol and waived the need for informed consent (UMC Utrecht Medical Research Ethics Committee 19-029/C).

### Patients

This retrospective observational study included patients aged ≥ 60 years with postoperative elevated troponin levels as detected by routine troponin monitoring within the first 3 days after noncardiac surgery between January 1, 2017, and December 31, 2018, at the University Medical Center Utrecht, a tertiary referral hospital. Patients admitted immediately after the procedure at the intensive care unit (ICU) or the cardiac care unit (CCU) for more than 2 days were not visited by the dedicated anesthesiologists and were therefore excluded from the analysis because follow-up in these patients was conducted by intensivists or cardiologists as part of our local protocol. Patients who died within 24 h or in whom further therapy was withheld directly after surgery were also excluded. Patients, who underwent surgery more than once within the study period, were included as a new case. However, in case the procedure was performed within 3 days after the previous procedure, only the first procedure was included in the analysis.

### Postoperative care

According to our local postoperative care protocol, cardiac troponin I (TnI) is measured in all noncardiac surgical patients aged ≥ 60 years once daily on the first three postoperative days during hospital admission. This protocol excludes ophthalmic and plastic surgery patients because of low risk of cardiac complications. Troponin elevation was defined as TnI above the clinical cutoff level, which is the lowest value measurable with a 10% coefficient of variation above the 99th percentile upper reference limit (Thygesen et al. 2018). Two different TnI assays were used over the years. This resulted in a clinical cutoff of TnI ≥ 60 ng L^−1^ (AccuTnI assay, Beckman Coulter, Brea, California, USA) from January 1, 2017, until May 16, 2018, and a clinical cutoff of high-sensitive TnI ≥ 18 ng L^−1^ (Unicel DxI 800, Beckman Coulter, Brea, California, USA) from May 17, 2018, until December 31, 2018.

Patients with elevated TnI were consulted within the first three postoperative days, and longer if indicated. Management of these patients was left to the judgement of the attending anesthesiologist, which was based on a local protocol. This protocol advises to optimize myocardial oxygen supply and demand, and to conduct follow-up of troponin and an electrocardiogram (ECG) in patients with TnI > 120 ng L^−1^ in case of no evident non-ischemic cause (e.g., sepsis, stress-induced cardiomyopathy) to rule out myocardial ischemia. This cutoff was based on two times the clinical cutoff of the assay used at that moment. Further, this protocol advises to consider consultation of a cardiologist, follow-up at the outpatient cardiac clinic, and prescription of antiplatelet therapy, statin, or beta-blockade. In patients with troponin > 600 ng L^−1^, i.e., more than ten times the clinical cutoff, a cardiology consultation is always recommended. Patients with mild troponin elevation were per protocol frequently not visited physically by the anesthesiologist because of a low risk of serious complications but were only followed up by TnI and ECG. A team of ten dedicated anesthesiologists with a particular interest in perioperative medicine were educated on the protocol.

### Data collection

All data were collected from electronic medical files. These data included patient characteristics, comorbidities, revised cardiac risk index (RCRI) (Lee et al. 1999), American Society of Anesthesiologists (ASA) physical status classification (American Society of Anesthesiologists 2014), metabolic equivalent task score (METs) (Kristensen et al. 2014), and surgical risk as defined by the RCRI, and by the European Society of Cardiology (ESC) and the European Society of Anaesthesiology (ESA) (Kristensen et al. 2014). Additionally, data on visits by the dedicated anesthesiologist, diagnostics, in-hospital postoperative complications, severity of complications according to the Clavien-Dindo classification (Dindo et al. 2004; Jammer et al. 2015), and in-hospital mortality were collected.

### Outcome

The primary outcome was early detected (i.e., ≤ 7 days after surgery) myocardial infarction as a result of consultation by the dedicated anesthesiologist. Myocardial infarction was defined as clinically diagnosed by the attending cardiologist. Secondary outcomes were overall detected complications, additional diagnostic tests advised by the dedicated anesthesiologist, treatment advised by the anesthesiologist, consultation of another medical specialist, and advised postoperative follow-up at the outpatient cardiac clinic. Potentially, a cardiologist could already have been consulted by the ward physician in patients with an elevated troponin or other cardiac complications, as the ward physicians were aware of the protocol. Therefore, we also recorded whether a cardiologist was consulted prior to or simultaneously with the visit by the dedicated anesthesiologists.

In addition to myocardial infarction, we assessed the occurrence of the following complications within 7 days after the procedure: arrhythmia diagnosed on 12-leads ECG or cardiac monitor, cardiopulmonary resuscitation, cerebrovascular accident (defined as radiologically confirmed ischemic or hemorrhagic stroke or transient ischemic attack), radiologically confirmed deep venous thrombosis and pulmonary embolism, sepsis as clinically diagnosed by the treating physician, pneumonia requiring antibiotics, respiratory failure requiring MCU (medium care unit) or ICU admission, acute kidney injury (AKI) defined as an increase in creatinine of 26.4 μmol L^−1^ or 25% from the preoperative creatinine value (Jammer et al. 2015), anemia defined as hemoglobin < 6.0 mmol L^−1^ (10 g dL^−1^, according to the Dutch guideline on blood transfusion(CBO 2011)), unexpected MCU or ICU admission, and unplanned reoperations. Additionally, length of hospital stay, mortality within 7 days, and the cause of death were assessed. Last, the Clavien-Dindo grade of the most severe complication within a week after surgery was recorded. A severe complication was defined as a Clavien-Dindo grade ≥ 3 as this involves complications requiring a surgical, endoscopic, or radiologic intervention; life threatening complications requiring care in a high-dependency or intensive care unit; or death. (Dindo et al. 2004; Jammer et al. 2015) Additionally, we assessed the final suspected etiology of the elevated troponin as proposed by the dedicated anesthesiologist.

### Statistical analyses

Baseline characteristics were compared dependent on the amount of troponin elevation (i.e., TnI 18–119 ng L^−1^, TnI 120–599 ng L^−1^, and TnI ≥ 600 ng L^−1^). These thresholds were chosen based on the thresholds as defined in our local protocol. The contribution of the dedicated anesthesiologist on the early detection of complications was assessed relative to the total number of complications. In addition, complications by their severity, length of stay, unexpected ICU or MCU admission, and death were assessed. The consultation rates by the dedicated anesthesiologists were evaluated, as were the number of ordered diagnostics, consulted medical specialties, and advised therapies. In order to determine whether care utility and complications were dependent on the height of troponin elevation, these were evaluated in subgroups of patients with different levels of troponin (i.e., TnI 18–119 ng L^−1^, TnI 120–599 ng L^−1^, and TnI ≥ 600 ng L^−1^).

Hemoglobin and creatinine measurements were missing in 103 and 204 patients, respectively. Since these variables were only used for descriptive statistics, we did not consider this an important source of bias and we did not impute the data. The statistical analyses were performed with R (Version 3.5.1–© 2018-07-02, R, Inc., for Windows) (R Core Team (2016)).

## Results

**Fig. 1:**
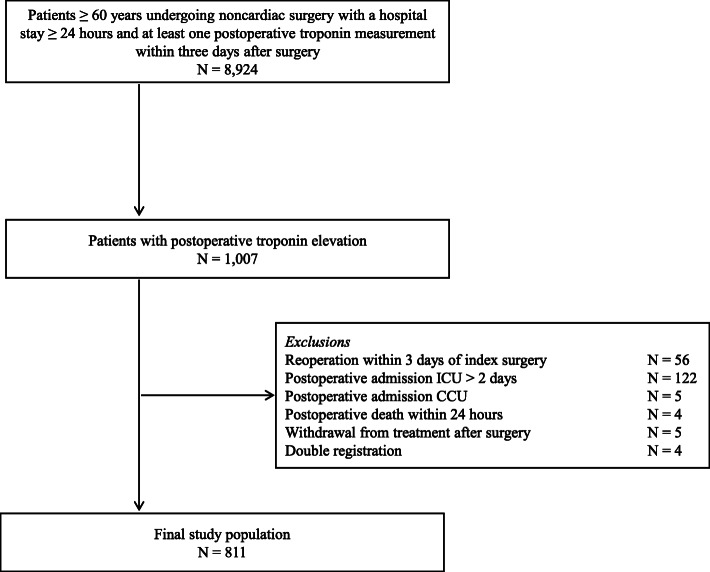
Patient inclusion. ICU, intensive care unit; CCU, cardiac care unit

Within the study period, 8924 patients underwent noncardiac surgery of whom 1007 patients (11.2%) had troponin elevation. Of these patients, 811 were eligible for inclusion (Fig. 1). Reason of exclusion was most often due to ICU admission for > 2 days (*n* = 122). Troponin was mildly elevated, i.e., 18–119 ng L^−1^ in 543 patients (67%); moderately elevated, i.e., 120–600 ng L^−1^ in 192 patients (24%); and highly elevated, i.e., ≥ 600 ng L^−1^ in 77 patients (9%).

Baseline characteristics are reported in Table 1. The median age was 74 years [IQR 68–80], and 62% of the patients were males. Most patients underwent general (28%) or vascular (24%) surgery, and surgery was emergent in 40% of the patients. There were more patients with a history of ischemic heart disease and peripheral vascular disease in the group with highly elevated troponin compared to the other two categories.

**Table 1:** Baseline characteristics dependent on the height of troponin elevation

	Overall	TnI 18–119 ng L^−1^	TnI 120–599 ng L^−1^	TnI ≥ 600 ng L^−1^
N	811	543	192	76
Age (median [IQR])	74 [68–80]	74 [68–80]	74 [68–79]	73 [69–79]
Males (%)	505 (62.3)	341 (62.8)	115 (59.9)	49 (64.5)
RCRI score (%)				
0	196 (24.2)	141 (26.0)	45 (23.4)	10 (13.2)
1	320 (39.5)	215 (39.6)	77 (40.1)	28 (36.8)
2	191(23.6)	120 (22.1)	45 (23.4)	26 (34.2)
3 or more	104 (12.8)	67 (12.3)	25 (13.0)	12 (15.8)
High-risk surgery (defined by RCRI) (%)	314 (38.7)	195 (35.9)	82 (42.7)	37 (48.7)
High-risk surgery (defined by ESC/ESA) (%)	124 (15.3)	77 (14.2)	35 (18.2)	12 (15.8)
History of ischemic heart disease (%)	235 (29.0)	145 (26.7)	58 (30.2)	32 (42.1)
History of congestive heart failure (%)	84 (10.4)	60 (11.0)	19 (9.9)	5 (6.6)
History of cerebrovascular disease (%)	201 (24.8)	132 (24.3)	48 (25.0)	21 (27.6)
Insulin dependent diabetes (%)	109 (13.4)	74 (13.6)	24 (12.5)	11 (14.5)
Preoperative creatinine > 2.0 mg dL^−1^ (%)	113 (13.9)	83 (15.3)	18 (9.4)	12 (15.8)
Arrhythmia (%)	185 (22.8)	136 (25.0)	42 (21.9)	7 (9.2)
ICD or pacemaker (%)	65 (8.0)	53 (9.8)	10 (5.2)	2 (2.6)
Valvular disease (%)	113 (13.9)	82 (15.1)	23 (12.0)	8 (10.5)
Peripheral vascular disease (%)	241 (29.7)	140 (25.8)	66 (34.4)	35 (46.1)
Hypertension (%)	516 (63.6)	347 (63.9)	119 (62.0)	50 (65.8)
Pulmonary disease (%)	210 (25.9)	142 (26.2)	54 (28.1)	14 (18.4)
Active malignancy (%)	255 (31.4)	172 (31.7)	60 (31.2)	23 (30.3)
Renal failure (%)	282 (34.8)	194 (35.7)	58 (30.2)	30 (39.5)
Diabetes mellitus (%)	189 (23.3)	129 (23.8)	41 (21.4)	19 (25.0)
ASA physical status (%)				
1	4 (0.5)	3 (0.6)	0 (0.0)	1 (1.3)
2	229 (28.2)	161 (29.7)	53 (27.6)	15 (19.7)
3	481 (59.3)	319 (58.7)	112 (58.3)	50 (65.8)
4	88 (10.9)	55 (10.1)	25 (13.0)	8 (10.5)
5	9 (1.1)	5 (0.9)	2 (1.0)	2 (2.6)
METS (%)				
1–3 METS	224 (27.6)	162 (29.8)	42 (21.9)	20 (26.3)
4–7 METS	373 (46.0)	263 (48.4)	79 (41.1)	31 (40.8)
8–10 METS	21 (2.6)	10 (1.8)	9 (4.7)	2 (2.6)
Unknown	193 (23.8)	108 (19.9)	62 (32.3)	23 (30.3)
Emergency surgery (%)	328 (40.4)	213 (39.2)	83 (43.2)	32 (42.1)
Surgical specialty (%)				
General	223 (27.5)	149 (27.4)	58 (30.2)	16 (21.1)
Gynecological	25 (3.1)	18 (3.3)	5 (2.6)	2 (2.6)
Head and Neck	78 (9.6)	54 (9.9)	15 (7.8)	9 (11.8)
Neurological	133 (16.4)	94 (17.3)	28 (14.6)	11 (14.5)
Orthopedic	104 (12.8)	71 (13.1)	26 (13.5)	7 (9.2)
Urological	57 (7.0)	42 (7.7)	9 (4.7)	6 (7.9)
Vascular	191 (23.6)	115 (21.2)	51 (26.6)	25 (32.9)
Locoregional and neuraxial anesthesia (%)	62 (7.6)	45 (8.3)	13 (6.8)	4 (5.3)

Troponin thresholds were chosen based on the thresholds as defined in the local protocol of the University Medical Center Utrecht (TnI 18–119 ng L^−1^, TnI 120–599 ng L^−1^, and TnI ≥ 600 ng L^−1^). Ischemic heart disease was defined as a history of myocardial infarction or previous revascularization, and a history of congestive heart failure was defined as an estimated left ventricular ejection fraction < 40%. Cerebrovascular disease was defined as a history of ischemic stroke, hemorrhagic stroke, or transient ischemic attacks. Renal failure was defined as a glomerular filtration rate (GFR) < 60 ml min^−1^ in the last 3 months)

*ASA* American Society of Anesthesiologists classification, *ESA* European Society of Anaesthesiology, *ESC* European Society of Cardiology, *ICD* implantable cardioverter defibrillator, *IQR* interquartile range, *METs* metabolic equivalent of task score, *RCRI* revised cardiac risk index, *TnI* troponin I

### Postoperative outcomes

A total of 804 complications occurred in 462 patients within 7 days after surgery (Table 2). The involvement of the dedicated anesthesiologist led to the diagnosis of 97 (12%) of these complications in 63 patients (13%). Postoperative complications were identified by the anesthesiologist in 18 patients (5%) with a TnI 18–119 ng L^−1^, 21 patients (13%) with a TnI 120–599 ng L^−1^, and 24 patients (33%) with a TnI ≥ 600 ng L^−1^. The anesthesiologists were involved in the discovery of 19 (59%) of 32 postoperative myocardial infarctions within 1 week after surgery. Besides cardiac ischemia, 76 (9.4%) patients developed an arrhythmia, but patients were also often diagnosed with noncardiac complications, including respiratory failure (8.9%), pneumonia (13.2%), and acute kidney injury (17.5%). Patients with higher troponin levels suffered from more complications, had more often an unexpected ICU or MCU admission, and had a longer median hospital length of stay. Ten patients (1%) died in the first week after surgery, and 251 patients (31%) had a complication graded as Clavien-Dindo ≥ 3 (Table 2).

**Table 2:** Postoperative outcomes dependent on the height of troponin elevation

	TnI 18–119 ng L^−1^		TnI 120–599 ng L^−1^		TnI ≥ 600 ng L^−1^	
*N*	543		192		76	
Complications ≤ 7 days	Total	By POA^c^	Total	By POA^c^	Total	By POA^c^
Myocardial infarction (%)	1 (0.2)	1 (0.2)	8 (4.2)	2 (1.0)	23 (30.3)	16 (21.1)
Arrhythmia (%)	46 (8.5)	3 (0.6)	22 (11.5)	0 (0.0)	9 (11.8)	3 (3.9)
CPR (%)	0 (0.0)	0 (0.0)	2 (1.0)	0 (0.0)	2 (2.6)	0 (0.0)
Cerebrovascular accident (%)	9 (1.7)	0 (0.0)	2 (1.0)	0 (0.0)	3 (3.9)	0 (0.0)
Deep venous thrombosis (%)	4 (0.7)	0 (0.0)	0 (0.0)	0 (0.0)	0 (0.0)	0 (0.0)
Pulmonary embolism (%)	10 (1.8)	0 (0.0)	5 (2.6)	0 (0.0)	3 (3.9)	1 (1.3)
Sepsis (%)	20 (3.7)	0 (0.0)	8 (4.2)	1 (0.5)	5 (6.6)	1 (1.3)
Pneumonia (%)	61 (11.2)	6 (1.1)	31 (16.1)	0 (0.0)	15 (19.7)	2 (2.6)
Respiratory failure (%)	33 (6.1)	1 (0.2)	25 (13.0)	6 (3.1)	14 (18.4)	1 (1.3)
Acute kidney injury^a^ (%)	59 (15.1)	1 (0.0)	33 (21.7)	0 (0.0)	14 (22.2)	4 (14.8)
Anemia^b^ (%)	198 (36.5)	6 (6.6)	87 (45.3)	28 (14.6)	42 (55.3)	14 (18.4)
Clavien-Dindo Classification (%)						
Grade 1	73 (13.4)		16 (8.3)		5 (6.6)	
Grade 2	206 (37.9)		67 (34.9)		25 (32.9)	
Grade 3a	11 (2.0)		10 (5.2)		1 (1.3)	
Grade 3b	26 (4.8)		9 (4.7)		2 (2.6)	
Grade 4a	74 (13.6)		47 (24.5)		27 (35.5)	
Grade 4b	19 (3.5)		7 (3.6)		8 (10.5)	
Grade 5	5 (0.9)		4 (2.1)		1 (1.3)	
No complications	129 (23.8)		32 (16.7)		7 (9.2)	
Unexpected ICU admission (%)	21 (3.9)		13 (6.8)		11 (14.5)	
Unexpected MCU admission (%)	91 (16.8)		5 (28.6)		33 (43.4)	
Reoperation (%)	50 (9.2)		19 (9.9)		8 (10.5)	
Length of stay (median [IQR])	7 [4–15]		9 [5–16]		11 [6–16]	
Mortality within 1 week (%)	5 (0.9)		4 (2.1)		1 (1.3)	

Troponin thresholds were chosen based on the thresholds as defined in the local protocol of the University Medical Center Utrecht (TnI 18–119 ng L^−1^, TnI 120–599 ng L^−1^, and TnI ≥ 600 ng L^−1^)

*CPR* cardiopulmonary resuscitation, *ICU* intensive care unit, *IQR* interquartile range, *MCU* medium care unit, *NA* not applicable, *TnI* troponin I

^a^Acute kidney injury (AKI) was calculated based on the KDIGO criteria. (1) AKI was based on 607 patients as no pre- and/or postoperative creatinine was available in 204 patients (25%)

^b^Anemia was defined as a hemoglobin < 6.0 mmol L^−1^ (10 g dL^−1^), which is based on 708 patients as hemoglobin was not measured in 103 patients (13%)

^c^Early detection of complication (within 3 days) through contribution of the dedicated team of anesthesiologist (POA: perioperative anesthesiologist)

### Postoperative consultation

A total of 509 patients (63%) received consultation by a dedicated anesthesiologist (Table 3). In 35 patients (7%), the anesthesiologists provided advice as recorded in the electronic medical file, without an actual physical visit. In patients who experienced at least one complication (*n* = 462), 161 patients (53%) were not visited by the dedicated anesthesiologist; in 18 patients (51%), only advice was provided without an actual physical visit by the anesthesiologist, 60 (59%) patients were visited once, and 99 (62%) patients were visited more than once. In 124 patients (24%), the cardiologist was already involved in patient care prior to or simultaneously with the dedicated anesthesiologist, and the cardiologist was consulted by the dedicated anesthesiologist in 155 patients (30%). In total, 187 patients (23%) were referred to the cardiac outpatient clinic for further follow-up after discharge.

**Table 3:** Consultations, interventions, and causes of troponin elevation assigned by the dedicated anesthesiologist, dependent on the height of troponin elevation

	TnI 18–119 ng L^−1^	TnI 120–599 ng L^−1^	TnI ≥ 600 ng L^−1^
*N*	543	192	76
Consultation (%)			
No visit, advice only	28 (5.2)	4 (2.1)	3 (3.9)
Only 1-time visit	186 (34.3)	90 (46.9)	39 (51.3)
> 1 visit	88 (16.2)	49 (25.5)	22 (28.9)
Any diagnostics ordered (%)	207 (38.1)	126 (65.6)	48 (63.2)
ECG	201 (37.0)	120 (62.5)	47 (61.8)
Echocardiography	3 (0.6)	19 (9.9)	26 (34.2)
CT angiography	2 (0.4)	3 (1.6)	4 (5.3)
Additional troponin	76 (14.0)	61 (31.8)	38 (50.0)
Other enzymes (i.e., CK-MB)	23 (4.2)	43 (22.4)	36 (47.4)
Other specialty consulted (%)			
Cardiology	39 (7.2)	65 (33.9)	51 (67.1)
Pulmonology	5 (0.9)	2 (1.0)	0 (0.0)
Other^a^	6 (1.1)	2 (1.0)	0 (0.0)
Cardiology already involved (%)	57 (10.5)	46 (24.0)	21 (27.6)
Any change in treatment (%)	55 (10.1)	47 (24.5)	29 (38.2)
Change in medication	24 (4.4)	26 (13.5)	24 (31.6)
Red blood cell transfusion	6 (6.6)	28 (14.6)	14 (18.4)
Follow up at outpatient cardiac clinic (%)	73 (13.4)	64 (33.3)	50 (65.8)
Cause of myocardial injury (%)			
Ischemic heart disease	6 (1.1)	12 (6.2)	28 (36.8)
Arrhythmia	22 (4.1)	8 (4.2)	0 (0.0)
Congestive heart failure	6 (1.1)	2 (1.0)	3 (3.9)
Pulmonary embolism	4 (0.7)	1 (0.5)	2 (2.6)
Pneumonia	7 (1.3)	4 (2.1)	0 (0.0)
Respiratory failure	11 (2.0)	9 (4.7)	1 (1.3)
Sepsis	9 (1.7)	6 (3.1)	2 (2.6)
Acute kidney injury	31 (5.7)	6 (3.1)	0 (0.0)
Anemia	15 (2.8)	8 (4.2)	4 (5.3)
Fluid overload	4 (0.7)	4 (2.1)	0 (0.0)
Hypertension	6 (1.1)	3 (1.6)	0 (0.0)
Perioperative hemodynamics^b^	66 (12.2)	37 (19.3)	9 (11.8)
Other^c^	15 (2.8)	8 (4.2)	3 (3.9)
Cause unknown	341 (62.8)	84 (43.8)	24 (31.6)

Troponin thresholds were chosen based on the thresholds as defined in the local protocol of the University Medical Center Utrecht (TnI 18–119 ng L^−1^, TnI 120–599 ng L^−1^, and TnI ≥ 600 ng L^−1^)

^a^Including nephrologist, hematologist, cardiothoracic surgeon, and geriatrician

^b^Including hypotension and tachycardia

^c^Including pericarditis, myocardial contusion, neurological conditions (e.g., subarachnoid hemorrhage), and fever

*ECG* electrocardiogram, *CT* computed tomography, *CK-MB* creatine-kinase isoenzyme, *MCU* surgical medium care unit, *PACU* post anesthesia care unit, *TnI* troponin I

There were more consultations by the dedicated anesthesiologists in patients with TnI ≥ 600 ng L^−1^ compared with the other two categories. In addition, a cardiologist was more frequently consulted (80% vs. 45% and 13% for patients with TnI ≥ 600 ng L^−1^ vs. TnI 120−599 ng L^−1^ and TnI 18–120 ng L^−1^, respectively) or already involved (33% vs. 32% and 19%, respectively) (Table 3).

Additional diagnostics were ordered by the anesthesiologist in 381 patients (75%). An ECG was most frequently ordered (73%), followed by additional troponin (34%) and other cardiac enzymes (20%). A change in treatment (i.e., a change in medication or red blood cell transfusion) was initiated by the anesthesiologist in 131 patients (16%), with 48 patients receiving blood products (6%). There were more diagnostic tests ordered and treatments initiated by the dedicated anesthesiologist in patients with higher troponin levels (Table 3).

In 449 (55%) patients, no clear cause of the troponin elevation was identified (Table 3). In 22% of patients, it was considered to be caused by unstable perioperative hemodynamics, including hypotension and tachycardia, followed by ischemic heart disease (9%) and AKI (7%).

## Discussion

This study evaluated the contribution of follow-up by dedicated anesthesiologists on postoperative care utility and early detection of complications in patients with elevated troponin after noncardiac surgery. The anesthesiologists were primarily responsible for the early detection of 12% of all postoperative complications within 1 week after surgery, with an especially large contribution (59%) to detection of myocardial infarctions.

### Literature

The current study found that routine postoperative troponin surveillance, supported by routine postoperative consultations by a dedicated anesthesiologist, can be used to detect postoperative complications at an early stage. Increasing rates of both cardiovascular, i.e., myocardial infarction and pulmonary embolism, and non-cardiac complications, i.e., sepsis, respiratory failure, renal failure, and anemia, were found in patients with elevated troponin in a dose-dependent manner. Substantially more patients with postoperative troponin elevation were consulted after implementation of a dedicated anesthesia team to conduct these visits (41% were consulted by cardiologists in the study by Van Waes et al. (2016) versus 63% in the current study).

Around 15% of patients with elevated troponin received a change in medication, which is in accordance with a previous study (van Waes et al. 2016). Six percent received red blood cell transfusion after involvement of the dedicated anesthesiologist, aiming at a hemoglobin level > 6 mmol L^−1^ (10 g dL^−1^). Interestingly, a previous study found that the cardiologist only advised red blood cell transfusion in 2% of patients with postoperative troponin elevation (van Waes et al. 2016).

### Clinical implications

Prediction of the risk of postoperative complications has been shown to be difficult using only preoperative parameters (Wijeysundera 2016). Although preoperative patient optimization and planning of perioperative care might have a larger effect on clinical outcome and costs than postoperative visits by trained anesthesiologist solely (Dexter and Wachtel 2014), we do believe there may be an additive beneficial effect provided by these visits. Ideally, one might argue that anesthesiologists should consult all patients receiving intermediate or high-risk surgery, but resources are scarce. By identifying patients at risk not only before surgery but again in the postoperative phase using additional data, resources can be directed towards where needed most. Troponin surveillance may be an efficient manner to conduct this postoperative selection (Devereaux et al. 2017; van Waes et al. 2016; Weber et al. 2013). Therefore, we focused on patients with elevated troponins to select those at risk of postoperative complications and to first determine the effect of this intervention, before unrolling it to a broader patient population.

The present study found that follow-up by dedicated anesthesiologists enabled early detection of 12% of all complications and 59% of all postoperative myocardial infarctions within 1 week after surgery. The experience of anesthesiologists with procedure-related complications in addition to their knowledge of the cardiopulmonary and central nervous system can provide a valuable contribution to postoperative care by surgeons and care by cardiologists in case of postoperative troponin elevation. In our institution, the resources needed for a dedicated team of anesthesiologists conducting postoperative visits in this group of patients are low. One dedicated anesthesiologist on call spends on average 1 h per day on screening and follow-up of patients with postoperative troponin elevation. In case of any emergent complications caught by this anesthesiologist, additional time is needed for extra diagnostics, consultation of other medical specialties, and follow-up. The remaining time is spent on clinical tasks at the outpatient clinic not evaluated in this study, such as preoperative assessment of patients and multidisciplinary meetings. In our center, no extra personnel needed to be employed to implement this program. In addition, a cardiologist was consulted in only 30% of patients instead of 41% as reported previously (van Waes et al. 2016). Although additional diagnostic procedures were performed, these were mostly low-cost such as an ECG, while the number of patients in whom a complication was diagnosed and medical treatment was changed, was substantial. Importantly, in a majority of the patients (55%) a clear cause of troponin elevation could not be identified. Therefore, treatment options currently are often limited. Prospective studies are necessary to further determine the potential effect of postoperative visits by dedicated anesthesiologist on patient outcome.

### Strengths and limitations

To the best of our knowledge, this is the first study reporting on the effect of postoperative anesthesia visits on resource utility and detection of postoperative complications in patients with postoperative troponin elevation. It was conducted in the non-cardiac surgery population, making these results generalizable to a large group of patients.

This study has some important limitations. First, because of the retrospective design of the study and limited number of patients, we were unable to estimate whether the implementation of routine postoperative anesthesia visits resulted in better patient outcomes. Second, two different troponin assays, i.e., normal TnI and the high sensitive TnI, were used during the inclusion period. As the second assay is more sensitive, this might have resulted in a higher incidence of troponin elevation. However, there were no differences in preoperative characteristics, but we did find more myocardial infarctions in the period the normal TnI assay was used together with more cardiology consultations, extra ordered diagnostics, and change in medication. Third, many patients were referred to the cardiac outpatient clinic for further follow-up, but often this was conducted in other institutions, since the UMC Utrecht is a tertiary referral hospital. This hampered our ability to track down diagnostics and interventions instituted during these follow-up visits. Fourth, there were some missing values for creatinine and hemoglobin. Incidence rates of postoperative anemia and AKI are probably overestimated as their markers were mostly missing in healthier patients. Fifth, because myocardial infarction was defined as a clinical diagnosis made by a cardiologist, the incidence of myocardial infarction may have been higher when it retrospectively would have been based on the 4th universal definition (Thygesen et al. 2018), as previously shown (Beattie et al. 2018; Devereaux et al. 2011; van Waes et al. 2016). Finally, only documented consultations by the anesthesiologist were recorded as consultation, which potentially explains why 37% of the patients did not receive a consultation. As part of the local protocol, anesthesiologists determined whether consultation was necessary based on clinical course reported in the electronic medical files and consultation of treating physicians by phone. We presume that in patients with elevated but low troponin levels (especially with TnI 19–120 ng/l), an ECG was remotely assessed by the anesthesiologist and discussed with the treating physician. In case of no abnormalities, nothing was documented in the electronical medical files, although in fact remote consultation did occur.

## Conclusion

Implementation of routine postoperative consultations by dedicated anesthesiologists resulted in early detection and treatment of complications in patients with postoperative elevated troponin levels.

## Data Availability

The datasets used and/or analyzed during the current study are available from the corresponding author on reasonable request.

## Abbreviations

AKI: Acute kidney injury
ASA: American Society of Anesthesiologists
CCU: Cardiac care unit
CK-MB: Creatine-kinase isoenzyme
CPR: Cardiopulmonary resuscitation
CT: Computed tomography
ECG: Electrocardiogram
ESA: European Society of Anaesthesiology
ESC: European Society of Cardiology
GFR: Glomerular filtration rate
ICD: Implantable cardioverter defibrillator
ICU: Intensive care unit
IQR: Interquartile range
MCU: Medium care unit
METs: Metabolic equivalent of task score
NA: Not applicable
PACU: Post anesthesia care unit
POA: Perioperative anesthesiologist
RCRI: Revised cardiac risk index
TnI: Troponin I
UMC: University Medical Center
